# What can we learn from wildlife sightings during the COVID‐19 global shutdown?

**DOI:** 10.1002/ecs2.3215

**Published:** 2020-08-06

**Authors:** Amanda J. Zellmer, Eric M. Wood, Thilina Surasinghe, Breanna J. Putman, Gregory B. Pauly, Seth B. Magle, Jesse S. Lewis, Cria A. M. Kay, Mason Fidino

**Affiliations:** ^1^ Department of Biology Occidental College Los Angeles California 90041 USA; ^2^ Arroyos & Foothills Conservancy Pasadena California 91102 USA; ^3^ Department of Herpetology and Urban Nature Research Center Natural History Museum of Los Angeles County Los Angeles California 90007 USA; ^4^ Department of Biological Sciences California State University Los Angeles California 90032 USA; ^5^ Department of Biological Sciences Bridgewater State University Bridgewater Massachusetts 02325 USA; ^6^ Department of Biology California State University San Bernardino California 92407 USA; ^7^ Urban Wildlife Institute Lincoln Park Zoo Chicago Illinois 60614 USA; ^8^ College of Integrative Sciences and Arts Arizona State University Mesa Arizona 85212 USA

**Keywords:** automated detection, backyard studies, citizen science, community science, coronavirus, long‐term ecological/ecosystem research network, multicity collaboration, SARS‐CoV‐2, urban ecology

## Abstract

During the worldwide shutdown in response to the COVID‐19 pandemic, many reports emerged of urban wildlife sightings. While these images garnered public interest and declarations of wildlife reclaiming cities, it is unclear whether wildlife truly reoccupied urban areas or whether there were simply increased detections of urban wildlife during this time. Here, we detail key questions and needs for monitoring wildlife during the COVID‐19 shutdown and then link these with future needs and actions with the intent of improving conservation within urban ecosystems. We discuss the tools ecologists and conservation scientists can use to safely and effectively study urban wildlife during the shutdown. With a coordinated, multicity effort, researchers and community scientists can rigorously investigate the responses of wildlife to changes in human activities, which can help us address long‐standing questions in urban ecology, inspire conservation of wildlife, and inform the design of sustainable cities.

## Introduction

The COVID‐19 pandemic has resulted in a worldwide shutdown of cities large and small. While drastically altering human lives, this massive shift in human activities—reduced motorized traffic, restricted travel and trade, shuttered businesses, and closed parks, beaches, and recreational areas—also has the potential to significantly impact wildlife. Early in the shutdown, images of wildlife in cities were common in news reports and social media, garnering increasing public attention and declarations of wildlife reclaiming urban habitats (Sahagun 2020). Yet, urban ecologists and community (citizen) scientists have long‐documented wildlife in cities, including mammals, insects, and other invertebrates, birds, and herpetofauna (Faeth et al. 2011, Ballard et al. 2017). Many urban‐dwelling species simply go overlooked by people amidst the daily chaos of city life, often only noticed by devices such as motion‐triggered cameras (Magle et al. 2019). In fact, new species are still being discovered in densely populated cities (Feinberg et al. 2014, Hartop et al. 2015). This long‐documented presence of wildlife in cities brings into question whether the reported wildlife sightings during the COVID‐19 shutdown are in fact indicative of a change in wildlife behavior.

While it is possible that wildlife claimed deserted streets and parks during the shutdown, the reported spike in sightings could simply be due to an increase in observations of wildlife that were always there (Garrard et al. 2008). After all, wildlife detections are not solely conditional on species presence in a given area—they must also be observed (MacKenzie et al. 2017). As city inhabitants followed stay‐at‐home orders and looked for ways to pass the time, did they take up wildlife watching as a new hobby? Did the already observant wildlife enthusiasts simply have more time to make and share their observations? Regardless of the cause, the relationship between humans and wildlife in urban settings changed during the shutdown, and understanding the reasons for that change could inform urban ecology and conservation.

This interdisciplinary question necessitates a global concerted effort across the biological and social sciences. We are in a moment in which we can not only investigate how urban wildlife and human–wildlife interactions change during a global shutdown, but also establish a rigorous, coordinated effort among cities worldwide to study urban ecology. This research is of ever‐increasing importance as urban ecosystems expand across the world (Grimm et al. 2008). Further, with likely additional waves of the pandemic (Xu and Li 2020) our efforts now will enable effective research into the future. Here, we outline several key research questions and identify what we can and cannot learn from this moment. We then outline tools for safely studying urban wildlife while under quarantine. Finally, we discuss the potential lasting impacts of the shutdown on wildlife. Addressing questions about wildlife activity during the pandemic is of great urgency as the conditions of the shutdown rapidly change. Urban ecological research conducted at this moment offers an unprecedented opportunity for engaging the public in wildlife conservation, providing baseline data in urban areas where detailed studies have not yet been accomplished, and informing policy on the design of cities that are safe for both wildlife and humans.

## Key Questions

Describing how wildlife and human–wildlife interactions change within urban areas during the shutdown could help us address long‐standing questions in urban ecology. By identifying key questions, urban ecologists can coordinate efforts across cities to quickly shift focus toward studying this transient phenomenon.

### How does the urban environment change as a result of the global shutdown and how do these changes affect urban ecology?

The global shutdown can change the urban environment in numerous ways that are relevant to wildlife, with reductions in traffic as well as air and noise pollution already documented (Isaifan 2020). Environmental variation within cities, including urban noise, vehicular traffic, and air and noise pollution, have long been known to be associated with shifts in wildlife behavior, dispersal, and survival (Kowarik 2011). Even the presence of humans can be stressful enough to elicit a response by wildlife, for instance by becoming more nocturnal to avoid peak times of human activity (Gaynor et al. 2018). However, many of these human‐mediated environmental changes are covaried or may interact, making it difficult to determine causal links (Faeth et al. 2011). With the global shutdown, there is an opportunity to not only isolate the effects of some of these factors but also investigate the same processes across multiple cities, increasing levels of replication (Magle et al. 2019). In particular, assessing responses to a lack of human presence could help us tease apart whether urban wildlife are responding more to the built environment itself or to human activities, informing wildlife conservation strategies such as the creation of buffer zones around urban green spaces (Fernández‐Juricic et al. 2001).

Yet, environmental changes during the shutdown may be detrimental to species that thrive in cities, the urban exploiters (sensu McKinney and Lockwood 1999). As restaurants and popular tourist locations close and food wastes become scarce in these areas, how will species that rely on human subsidies respond? For instance, the closing of stadiums could result in a loss of foraging grounds for urban bats (Schoeman 2016). Animals may either be unable to find food or will be forced to shift their behaviors to forage in more residential or natural areas. This could become a quasi‐ecological trap, where urban wildlife may expose themselves to increased incidences of human–wildlife conflicts or predation by either human commensals or other predators as a result of reduced antipredator responses that have evolved in the urban environment (Magle et al. 2005). Quantifying the negative impacts of the shutdown on urban wildlife will help us understand the complex relationships that urban exploiters and adapters have developed with humans (Kark et al. 2007).

### How do different policies and approaches to the shutdown across cities affect wildlife?

One unique aspect of the shutdown is that we can assess how different approaches to modifying and enforcing changes in human behavior within cities may lead to different outcomes for wildlife. For example, cities that kept natural spaces open during the shutdown may inadvertently have contributed to negative impacts on wildlife if more people visited these sites than usual. Increased human activity in parks and along nature trails is associated with behavioral fear responses (Putman et al. 2017), which can often lead to low body conditions and poor health. How species respond to the shutdown across municipalities with different regulations could help inform policy within cities or management of urban green spaces to minimize stresses on wildlife.

### How does restricted global travel and trade impact urban wildlife?

Because the movement of humans and goods within and among cities leads to the export, either active or passive, of species outside their native ranges (Francis and Chadwick 2015), reduced international travel and shipping of goods during the shutdown will at least temporarily slow the movement and establishment of nonnative species in cities. This decrease in movement of wildlife can impact urban species in a variety of ways, including community composition and shifting ecological interactions between native and nonnative species (Kowarik 2011). Further, because many diseases that affect wildlife also move through wildlife trade (Karesh et al. 2005), urban wildlife may also experience reduced disease transmission. Reductions in the spread of nonnative species may be particularly large and long‐lasting if governments take additional steps to restrict wildlife trade to prevent the spread of zoonotic diseases (Yuan et al. 2020). If decreases in global travel and trade are associated with decreased movement of nonnative species and wildlife diseases during the shutdown, this could establish a roadmap for preventing the spread of these species in the future.

### Will we be able to detect population genetic or urban evolutionary responses to the shutdown?

Evolutionary responses to the shutdown would depend on how long shutdowns modify human activities and to what degree. Notably, with decreased traffic and human activity, urban wildlife may be more successful dispersers (Gunson et al. 2011), allowing for increased gene flow among isolated populations. If this short‐term shutdown is reflected in population genetic structure, it could provide insight into future management strategies that could promote gene flow among isolated urban populations. Further, how species respond to these temporary environmental changes could help us understand the extent to which local adaptation has already occurred in urban environments, allowing us to disentangle plasticity from evolutionary adaptations (Johnson and Munshi‐South 2017). While the shutdown may only have a minor impact on evolution in urban wildlife, it still provides an opportunity to study evolutionary responses among urban wildlife.

### How have human–wildlife interactions changed during the shutdown and how might that impact humans?

While the COVID‐19 pandemic is not how researchers wanted to assess the impact of modified human activity on urban wildlife, the stories of urban wildlife sightings being shared around the world illustrate the power that human–wildlife interactions have to heal during a time of extreme stress due to social isolation, illness, lost jobs, and uncertainty. Not only do human–wildlife interactions have multiple psychological benefits (Kowarik 2011), but these first‐hand experiences with wildlife during the pandemic could act to reshape city dwellers' understandings of and relationships with local species (Genovart et al. 2013). As most people live in cities (Grimm et al. 2008), urban wildlife sightings may be the kind of nature the majority of Earth's inhabitants regularly experience. Yet, not all human–wildlife interactions are positive and the same interactions could be interpreted differently by different people. Further, urbanization alters the nature of human–wildlife interactions. For instance, urbanization increases the transmission of zoonotic diseases (Hassell et al. 2017), leading to negative perceptions of wildlife. Communicating the relative risks of human–wildlife interactions without compromising messages about species conservation will thus be important (Decker et al. 2010). Consideration of the impacts of human–wildlife interactions on humans during the shutdown need to be considered from multiple lenses within diverse city populations.

### What if any will be the lasting impacts of the shutdown on urban wildlife?

Relative to many ecological and evolutionary timescales, the impact of the shutdown may only be a blip on the radar. Perhaps an uptick in movement of urban wildlife will temporarily boost gene flow among wildlife populations or help species re‐establish on isolated islands of urban green space, resetting some equilibria (Ryan et al. 2020). If changes in body condition, reproduction, and survival of wildlife occur because animals are better able to exploit the urban environment during the shutdown, this might lead to population‐, community‐, or ecosystem‐level processes in the following year such as increased population sizes or altered trophic dynamics (Shochat et al. 2006). Because there may likely be a lag in how urban wildlife populations respond to the quarantine, researchers should continue to monitor populations after the shutdown ends to evaluate these longer‐term patterns. On the other hand, if the shutdown leads to lasting shifts in human activities, such as less traffic as more people work from home, then perhaps the global quarantine will have longer lasting effects on urban wildlife.

## Urban Ecology Toolbox During a Global Shutdown

**Fig. 1:**
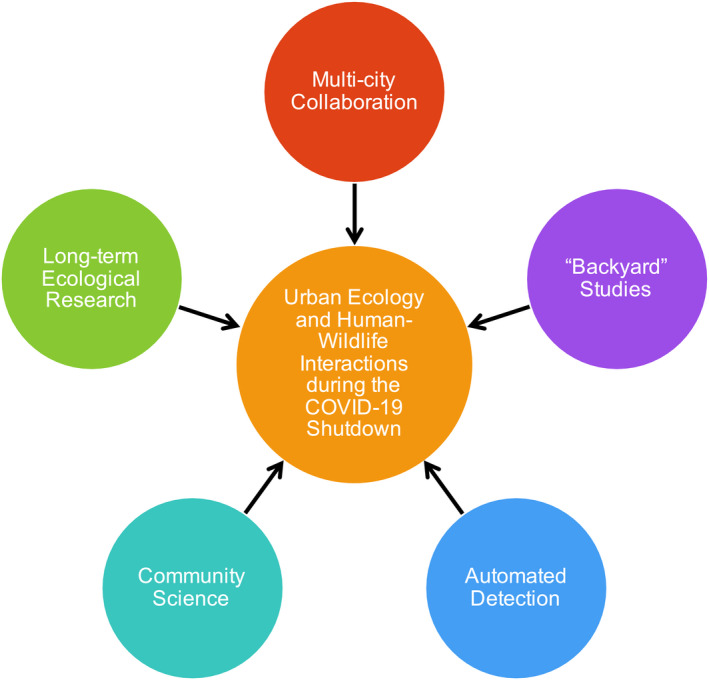
Conceptual framework for studying changes in urban wildlife and human–wildlife interactions as a result of the COVID‐19 shutdown of cities worldwide.

Like others, many urban ecologists are subject to the stay‐at‐home orders of the shutdown—so how do they safely and effectively assess changes in urban wildlife while being relegated to their neighborhoods? Fortunately, urban ecologists already have a wealth of tools at their disposal that leverage long‐term ecological research, community science, automated data collection, and virtual networking across multiple cities (Fig. 1).

### Community science

One of the best tools ecologists have for teasing apart changes in wildlife activities from observer behavior during the shutdown is community science. Biodiversity databases, such as iNaturalist (www.inaturalist.org) and eBird (www.ebird.org), have data that stretch well before the shutdown enabling comparisons with long‐term trends and have worldwide coverage, especially within cities. Notably, the 5th annual City Nature Challenge, a global competition among cities to document urban wildlife, was held during the pandemic with over 41,000 people from 244 cities around the world recording more than 815,000 nature observations (www.citynaturechallenge.org). These data could be used to assess how wildlife observations and wildlife observers differ from previous and future years. Beyond simple presence data, community science observations can also be used to study differences in ecological risks, such as predation or parasitism, across varying urban habitats (Putman et al. 2020). Importantly, outreach efforts have the potential to attract new community scientists and engage them in research and conservation into the future (Lewandowski and Oberhauser 2017). However, as urban ecologists call upon the public to engage in community science, scientists and volunteers alike need to ensure they are following all government recommended guidelines for social distancing, while also engaging in wildlife distancing to ensure the safety of wildlife (e.g., maintain a safe distance, do not alter habitat, and do not cause stress to wildlife or alter behavior).

### Long‐term urban ecological research

The question of how wildlife respond to the global quarantine requires a careful assessment of wildlife sightings before, during, and after the shutdown, especially in the context of long‐term trends and typical seasonal variation. Data collected through long‐term ecological/ecosystem research networks (LTERs) in cities will be integral to assessing this question, because these datasets provide crucial baseline estimates on urban ecosystems from which to compare novel wildlife sightings using standardized practices. While the majority of LTERs are in more natural areas, there are a few notable sites within cities (e.g., Central Arizona‐Phoenix LTER, Baltimore LTER; Brazel et al. 2000). Even in cities where there are no formal LTERs, other long‐term studies and even historic museum collections, which themselves are centered in urban areas, can be useful sources of baseline data (Lister et al. 2011). However, researchers may face obstacles to maintain data collection if they are prohibited from visiting field sites. Ensuring that urban ecologists can engage in this research needs to be a priority, particularly through establishment of government or nonprofit rapid‐access funding lines (e.g., NSF RAPID), fast‐tracking of permitting processes, and maintenance of field site access. Even if baseline data are not yet available within a city, which may likely be the case for many cities, researchers could establish studies now, provided it is safe to do so, to collect data during and post‐shutdown. There are now a variety of techniques to combine seemingly disparate data sources of varying data quality to more robustly evaluate ecological patterns (Pacifici et al. 2017). Further, establishing these studies now may be useful in anticipation of additional shutdowns during subsequent waves of the pandemic.

### Multicity collaborations

Unlike any other time in recent history, ecologists have an opportunity to assess urban ecology and human–wildlife interactions across multiple cities undergoing similar shifts in human activities, allowing for multicity comparisons and collaborations. One of the primary challenges that urban ecologists face is disentangling city‐specific influences on patterns of urban wildlife occurrence from broader ecological processes, given that each city has its own unique structure and history (Magle et al. 2019). Multicity research networks, such as the Urban Wildlife Information Network, have illustrated that species' responses to urbanization depend on landscape‐scale differences in green space availability and housing density among cities (Fidino et al., *unpublished manuscript*). However, such collaborations are rare and multicity comparisons can be difficult without standardized methodologies. Multicity research collaborations therefore provide a crucial framework for investigating the impacts of the COVID‐19 shutdown by standardizing methods across cities experiencing similar decreases in human activities.

### Automated detection

With some researchers unable to visit field sites, the shutdown further highlights the need for more urban ecological research that uses automated, remote monitoring devices, such as camera traps, bioacoustics devices, data loggers, and autonomous vehicles or drones. Research projects already implementing these devices prior to the shutdown will be invaluable for studying before and after effects. Laboratories that use these devices for research in more natural spaces or for projects that were canceled due to the shutdown could redirect their automated devices to study urban wildlife in the interim. Further, collaborations among researchers in different cities using the same devices could prove beneficial. If nothing else, establishing more studies using automated devices will be useful for studying future changes to urban environments.

### “Backyard” studies

**Fig. 2:**
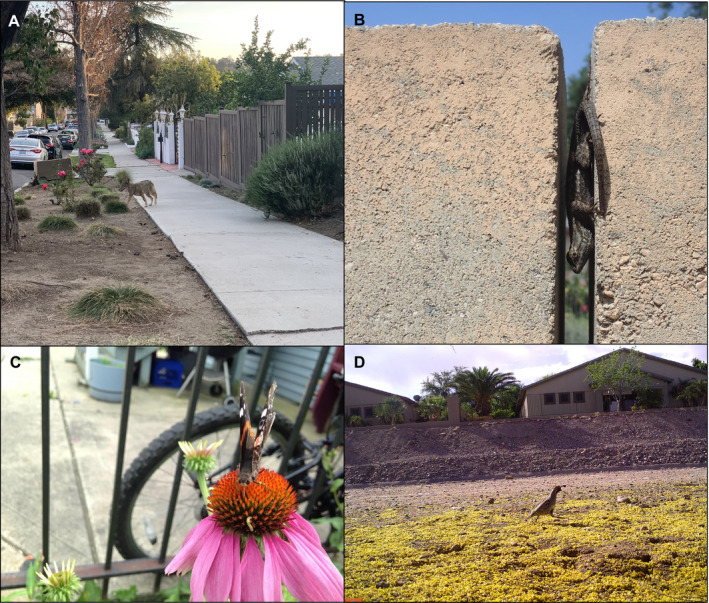
Variation in urban wildlife and urban environments illustrated across the yards of urban ecologists. (A) Coyote (*Canis latrans*) on sidewalk, Los Angeles, California, USA (iNaturalist observation number: 38627446; credit: A.J. Zellmer); (B) western fence lizard (*Sceloporus occidentalis*) in wall microhabitat, Claremont, California, USA (credit: B.J. Putman); (C) red admiral butterfly (*Vanessa atalanta*) on a purple coneflower (*Echinacea purpurea*), Chicago, Illinois, USA (credit: M. Fidino); and (D) Gambel's quail (*Callipepla gambelii*), Phoenix, Arizona, USA (credit: J.S. Lewis).

Even local‐scale studies in a researcher's yard or neighborhood, which have traditionally been neglected in the urban ecology literature, may be insightful (e.g., Cunningham 1960). A single backyard can contain multiple individuals of the same species, especially for small‐bodied, dispersal‐limited organisms such as many herpetofauna and invertebrates. By focusing on localized populations, researchers can collect detailed behavioral data to understand space use, time budgets, social structure, and repeatability of behavior. Backyard studies could also be scaled up for more regional or global analyses through collaborations distributed across the backyards of urban ecologists, undergraduates partaking in online biology courses, or community scientists in different neighborhoods (Fig. 2), allowing for detailed investigations into fine‐scale factors influencing urban wildlife traits.

## Conclusions

Investigating urban wildlife responses to the COVID‐19 shutdown with a globally coordinated effort can help inform long‐standing questions in urban ecology and provide opportunities for engaging the public in urban wildlife research and conservation. Even if there are no lasting changes in how our cities move and function, the wildlife sightings of the shutdown will be preserved in our documented research, in community science databases, and in the memories of those who gained a new appreciation of wildlife during an unprecedented time. Those data can provide a roadmap for interpreting urban ecology research, inspiring conservation, and designing sustainable cities in the future. If nothing else, the shutdown will give us a chance to ruminate on what wildlife‐friendly cities might look like, and how our science can point the way toward them.
